# Ozone and Temperature May Hinder Adaptive Capacity of Mediterranean Perennial Grasses to Future Global Change Scenarios

**DOI:** 10.3390/plants12030664

**Published:** 2023-02-02

**Authors:** Samuel Prieto-Benítez, Raquel Ruiz-Checa, Ignacio González-Fernández, Susana Elvira, Isabel Rucandio, Rocío Alonso, Victoria Bermejo-Bermejo

**Affiliations:** 1 Ecotoxicology of Air Pollution, Environmental Department CIEMAT, 28040 Madrid, Spain; 2 Spectroscopy, Technology Department CIEMAT, 28040 Madrid, Spain

**Keywords:** *Agrostis castellana*, *Festuca iberica*, *Festuca indigesta* subsp. *curvifolia*, *Stipa tenacissima*, gas exchange, plant growth, plant nutrients, O_3_-sensitivity, climate warming

## Abstract

Climate warming is recognized as a factor that threatens plant species in Mediterranean mountains. Tropospheric ozone (O_3_) should also be considered as another relevant stress factor for these ecosystems since current levels chronically exceed thresholds for plant protection in these areas. The main aim of the present study was to study the sensitivity of four Mediterranean perennial grasses to O_3_ and temperature based on plant growth, gas exchange parameters (photosynthesis—A, stomatal conductance—g_s_, and water use efficiency—WUE), and foliar macro- (N, K, Ca, Mg, P, and S) and micronutrients (B, Cu, Fe, Mn, Mo, and Zn) content. The selected species were grasses inhabiting different Mediterranean habitats from mountain-top to semi-arid grasslands. Plants were exposed to four O_3_ treatments in Open-Top chambers, ranging from preindustrial to above ambient levels, representing predicted future levels. Chamber-less plots were considered to study the effect of temperature increase. Despite the general tolerance of the grasses to O_3_ and temperature in terms of biomass growth, WUE and foliar nutrient composition were the most affected parameters. The grass species studied showed some degree of similarity in their response to temperature, more related with phylogeny than to their tolerance to drought. In some species, O_3_ or temperature stress resulted in low A or WUE, which can potentially hinder plant tolerance to climate change. The relationship between O_3_ and temperature effects on foliar nutrient composition and plant responses in terms of vegetative growth, A, gs, and WUE constitute a complex web of interactions that merits further study. In conclusion, both O_3_ and temperature might be modifying the adaptation capacity of Mediterranean perennial grass species to the global change. Air pollution should be considered among the driving favors of biodiversity changes in Mediterranean grassland habitats.

## 1. Introduction

Tropospheric ozone (O_3_) is one of the most relevant air contaminants due to its high toxicity, wide distribution, and its greenhouse effect [1,2,3,4]. Transportation and industrial activity are the main anthropogenic sources of O_3_ precursors. However, these pollutants are transported by air masses and can react with precursor emissions from natural sources causing large O_3_ surface levels in rural and forested areas far away from anthropic activities [5,6].

The photochemical formation and persistence of O_3_ is favored under Mediterranean climatic conditions, causing an important air pollution problem in the Mediterranean Basin [4,6,7,8]. The current O_3_ levels in the Iberian Peninsula chronically exceed the threshold levels for plant protection established by the UNECE Air Convention [9] and the EU Air Quality Directive (2008/50/EC) [10]. This problem is even more acute in the Mediterranean mountains [11,12].

Normal plant development is greatly impaired due to ozone [13,14,15]. Its oxidative capacity affects plants at different scales, from metabolic to ecosystem, causing effects on growth and biomass production [16,17], nutrient imbalances [18,19,20], or reproductive capacity [3,21], and finally affecting the structure and biodiversity of plant communities [22,23].

The higher levels of this pollutant in the Mediterranean area contrast with the fact that the Mediterranean Basin is considered a global biodiversity hotspot [24,25,26]. At the Spanish Central System mountain range, the spring–summer snow-free zones above the tree line harbor high levels of taxonomic diversity [27,28,29], including plant communities where grasses are dominant. Furthermore, in the Mediterranean Basin, grasses dominate large arid or semiarid areas [30].

The experimental evidence on the O_3_ sensitivity of Mediterranean grassland species varies greatly between community types. Mediterranean annual pasture species growing at lower altitudes have been the most extensively explored. In terms of biomass growth, Fabaceae species are generally more sensitive to O_3_ than Poaceae [31,32] but tolerant legume species have also been identified [33]. Annual grasses also responded to O_3_ by increasing leaf senescence or decreasing their life-span [34,35]. Regarding perennial grass species, most studies have been focused on species from more temperate climates [36,37,38], with less attention to species characteristics of the Mediterranean area [39]. Alpine and mid-elevation perennial temperate grass species have shown a wide variety of O_3_ responses, from tolerant to carry over O_3_-effects on above-ground biomass [38,40,41,42]. However, there has been no study addressing the O_3_ effects on Mediterranean perennial alpine species.

Plant communities at the top of Spanish Central System Mountains are very valuable for their high biodiversity, with a great number of endemic and relict species, and for being the southern limit of other more widely distributed species. In addition, within these communities, there are grass species which are of great economic interest in the region, since they are the basis for livestock feeding, especially during the summer season [43,44]. These summits are characterized by poor acid soils covered by snow during winter and with a high soil moisture deficit during the summer season, inhabited by the xerophytic grass species *Festuca indigesta* subsp. *curvifolia* (hereafter *F. indigesta*) [44]. The *Cervunal*, a neighboring community dominated by *Nardus stricta*, occupies patches with higher soil humidity [44]; *Festuca iberica* and *Agrostis castellana* are two other *grass* species characteristic of the *Cervunal* habitat. There is another interesting grassland community dominated by the esparto grass *Stipa tenacissima*, that in the Central Iberian Peninsula lives at a lower elevation but it can be found at 1400 m.a.s.l. in the southern mountain ranges. This “esparto” grass lives in drier and warmer habitats, facilitates the biological soil crusts and vascular plant establishment in semiarid habitats, and it is an important species for the local economy due to its use as raw material for the manufacture of clothing, footwear, basketry, or paper pulp production [45,46]

In this study, the O_3_ sensitivity of *A. castellana*, *S. tenacissima*, *F. iberica*, and *F. indigesta* was tested in an O_3_ fumigation experiment using Open Top Chambers (OTCs). The four species, belonging to three different tribes or subtribes within the *Pooideae* subfamily [47], together with their different habitats and water requirements, make them a good representation for testing the tropospheric O_3_-response of Mediterranean perennial grasses.

The main aim of the present study was to assay the O_3_-effect on Mediterranean perennial grasses according to plant growth, gas exchange (photosynthesis (A), stomatal conductance (g_s_), and water use efficiency (WUE)), and leaf composition considering macro- (N, K, Ca, Mg, P, and S) and micronutrients (B, Cu, Fe, Mn, Mo, and Zn). Based on previous results on the O_3_-response of Mediterranean annual grasses growing at lower elevations, we hypothesize that Mediterranean perennial grasses would present some tolerance to the pollutant based on growth parameters. However, we expected more subtle effects at a physiological scale on gas exchange parameters and foliar nutrients that might challenge their competitive capacity and compromise their tolerance to other stresses frequent in their habitat.

Taking advantage of the OTC experimental design, the effects of temperature increase were also tested at the same time as the O_3_ effect following [48]. Climate warming is one of the most important threats for plant species from the Mediterranean basin mountains, including the Spanish Central System mountain range [49]. We expect that species adapted to drier environments (*S. tenacissima* and *F. indigesta*) will be more tolerant to both, O_3_ and temperature increase, than species from moister areas. Specifically, we aimed to answer the following questions: (i) can we find any indicator of O_3_ effects on Mediterranean perennial grasses considering plant growth, leaf nutrients, or gas exchange?; (ii) might any of the O_3_ and temperature sensitive parameters compromise the long-term survival of the species under the global change scenarios for the Mediterranean Basin?; (iii) is water stress tolerance linked to O_3_ tolerance or temperature resistance?; and (iv) is the O_3_ sensitivity of species more related to water deficit tolerance or to their phylogenetic signature?

## 2. Results

### 2.1. Ozone Exposure and Temperature Increase

Accordingly with the experimental design, the lowest O_3_ concentration (nL L^−1^) was found in the CFA treatment (Table 1), with an O_3_ filtration efficiency of 47.5% compared with NFA. Non-filtered air OTCs and AMB plots showed comparable O_3_ levels throughout the fumigation period, the pollutant levels inside the OTC being only 5% lower than the AMB plots. Plants under the NFA++ treatment were the most exposed to the pollutant, with average O_3_ concentrations 37.5% higher than NFA levels, which caused the highest AOT40 values close to 15,000 nL L^−1^ h, above 10,000 nL L^−1^ h higher than AMB and NFA. NFA+ O_3_ concentration values fell in between NFA and NFA++ treatments, with cumulative AOT40 values in the range of 8500 nL L^−1^ h. During the daylight hours from 7:00 to 15:00 (GMT), OTCs compared with AMB plots presented an increase in the mean air T by 20.6% which implied an increment of around 5 °C inside the OTCs, also a reduction in RH by 4% was found; both caused an increase in the water Vapor Pressure Deficit (VPD) by 30.5% (Table 2).

### 2.2. Vegetative Growth

**Figure 1:**
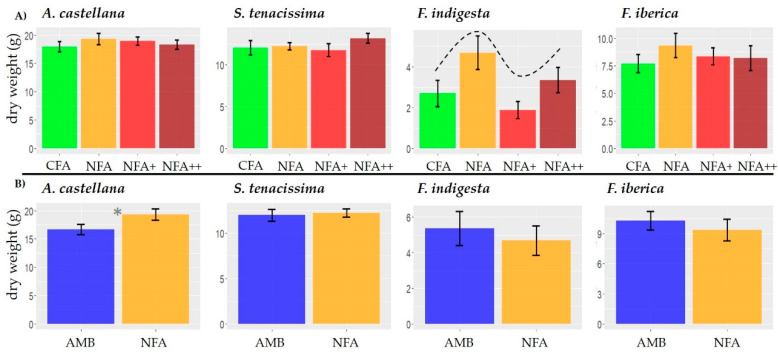
Vegetative aboveground growth response to O_3_ (**A**) and temperature increase (**B**) for *A. castellana*, *S. tenacissima*, *F. indigesta*, and *F. iberica.* Bars denote mean ± SE. Dotted lines denote marginally significant trends. Grey asterisk denotes marginally significant differences.

The vegetative growth of all the species was relatively O_3_ tolerant (Figure 1A). Table S3 shows the lineal effect (LE), quadratic effect (QE), and cubic effect (CE) contrasts for each species. A marginally CE of O_3_ was found in *F. indigesta* (t_6_ = 2.22, *p* = 0.07) where NFA and NFA++ had the highest growth. However, in *A. castellana* (t_66_ = −1.18, *p* = 0.24 for a QE), *S. tenacissima* (t_64_ = −1.12, *p* = 0.27 for a LE), and *F. iberica* (t_6_ = 0.37, *p* = 0.48 for a QE) no significant O_3_ effect was detected. The temperature increase did not affect vegetative growth for either *F. indigesta* (F_1, 2_ = 0.11; *p* = 0.77), *F. iberica* (F_1, 2_ = 0.01; *p* = 0.94), or *S. tenacissima* (F_1, 32_ = 0.1; *p* = 0.76) (Figure 1B, Table S4). However, the vegetative growth was marginally higher in NFA than AMB in *A. castellana* (F_1, 32_ = 3.95; *p* = 0.055). The structure of the random part of the model for each variable and species is presented in the Table S5.

### 2.3. Gas Exchange

**Figure 2:**
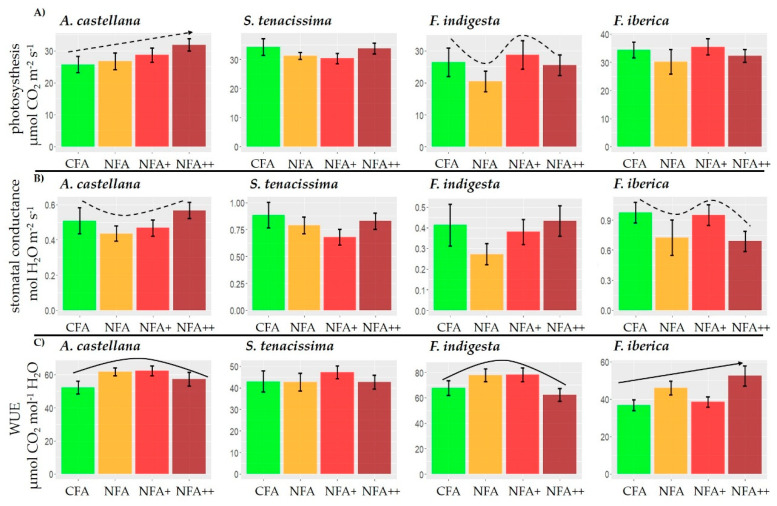
Ozone effect on gas exchange parameters: photosynthesis (A, μmol CO_2_ m^−2^s^−1^) (**A**), stomatal conductance (g_s_, mol H_2_O m^−2^s^−1^), (**B**) and water use efficiency (WUE, μmol CO_2_ mol^−1^ H_2_O) (**C**) for *A. castellana*, *S. tenaccissima*, *F. indigesta*, and *F. iberica*. Bars denote mean ± SE. O_3_ treatments: charcoal-filtered air (CFA), non-filtered air (NFA), non-filtered air + 20nL L^−1^ of O_3_ (NFA+), and non-filtered air + 40nL L^−1^ of O_3_ (NFA ++) treatments. Solid and dotted lines denote significant and marginally significant trends, respectively.

Table S3 shows the lineal effect (LE), quadratic effect (QE), and cubic effect (CE) contrast for each species. Considering the O_3_ effects on net photosynthesis (Figure 2A), a marginally significant LE was observed in *A. castellana* (t_20_ = 1.93, *p* = 0.07). However, *F. indigesta* presented a marginally significant CE with maximum values under NFA+ (t_16_ = −2.03, *p* = 0.06); a similar response was found in *F. iberica* although it was not significant (t_26_ = −1.29, *p* = 0.21). The observed response of A in *S. tenacissima* was neither significant (t_30_ = 1.61, *p* = 0.12 for a QE).

Regarding g_s_ (Figure 2B), three of the four species presented a similar concave trend in response to O_3_, with a greater stomatal opening under the lowest and highest O_3_ concentrations (CFA and NFA++ treatments). In *A. castellana*, NFA and NFA+ treatments were marginally lower than CFA and NFA++ (t_20_ = 1.79 *p* = 0.09); but even though *S. tenacissima* and *F. indigesta* showed the same concave pattern, they were not significant (t_30_ = 1.42, *p* = 0.17; t_16_ = 1.52, *p* = 0.15, respectively, for a QE). Contrary to conductance response in the other species, a marginally CE was observed in *F. iberica* (t_26_ = −1.75, *p* = 0.09).

Consistently with g_s_ values, the most observed WUE response pattern to the increased O_3_ levels was a convex QE (Figure 2C). In *A. castellana* WUE was higher in NFA and NFA+ than in CFA and NFA++ (QE t_20_ = −2.22 *p* = 0.04); a similar effect was observed in *F. indigesta* (t_16_ = −2.37, *p* = 0.03). Although in *S. tenacissima* the observed trend approached a convex response, the CE obtained the lowest *p*-value and was not significant (t_30_ = −0.97, *p* = 0.34). As happened with g_s_, the WUE behavior in *F. iberica* was different from the rest of species with a significantly increasing LE (t_26_ = 2.26, *p* = 0.03).

**Figure 3:**
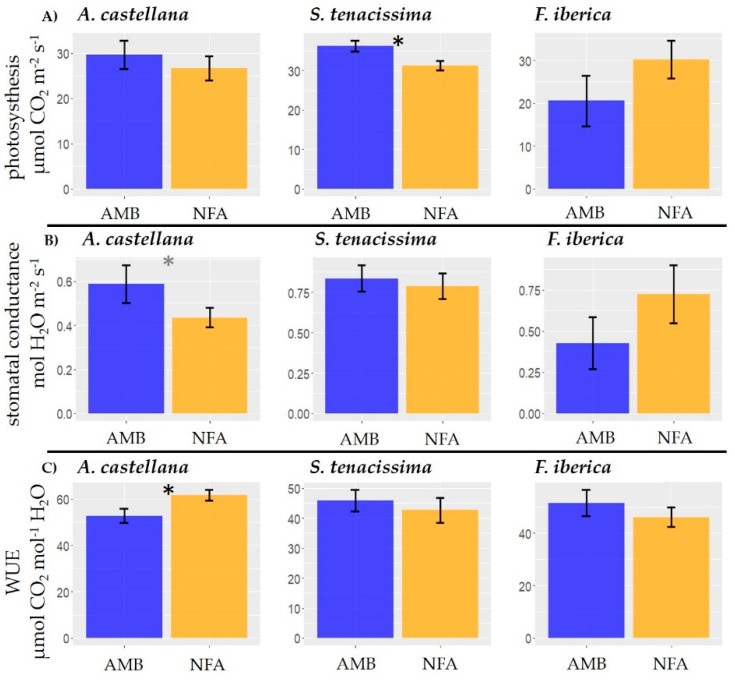
Temperature effects on gas exchange parameters: photosynthesis (**A**, μmol CO_2_ m^−2^s^−1^) (**A**), stomatal conductance (g_s_, mol H_2_O m^−2^s^−1^) m (**B**) and water use efficiency (WUE, μmol CO_2_ mol^−1^ H_2_O) (**C**) for *A. castellana*, *S. tenaccissima*, and *F. iberica.* Bars denote mean ± SE. Temperature treatments: low temperature (AMB plots), high temperature (NFA OTC). Black and grey asterisk denote significant and marginally significant differences, respectively.

The temperature increase caused different effects on the gas exchange parameters depending on the species (Figure 3, Table S4). Photosynthetic activity was reduced in *S. tenacissima* plants grown inside the NFA OTCs compared with the AMB plots (F_1, 7_ = 7.24, *p* = 0.02), but no temperature effect was detected in *F. iberica* (F_1, 7_ = 1.73, *p* = 0.23) and *A. castellana* (F_1, 9_ = 0.53, *p* = 0.48). Regarding g_s_, there was no difference between AMB and NFA in *F. iberica* (F_1, 7_ = 1.75, *p* = 0.23) and *S. tenaccissima* (F_1, 14_ = 0.17, *p* = 0.69). However, in *A. castellana* g_s_ was marginally higher under the lower temperatures of the AMB plots (F_1, 9_ = 3.59, *p* = 0.09). On the contrary, in the latter species, WUE was larger in NFA under the higher temperature values than in the AMB plots (F_1, 9_ = 5.65, *p* = 0.04). No differences due to temperature were found for WUE in *F. iberica* (F_1, 7_ = 0.75, *p* = 0.41) and *S. tenacissima* (F_1, 14_ = 0.49, *p* = 0.49). The structure of the random part of the model for each variable and species is presented in the Table S5.

### 2.4. Nutrients and Nutrient Ratios

**Figure 4:**
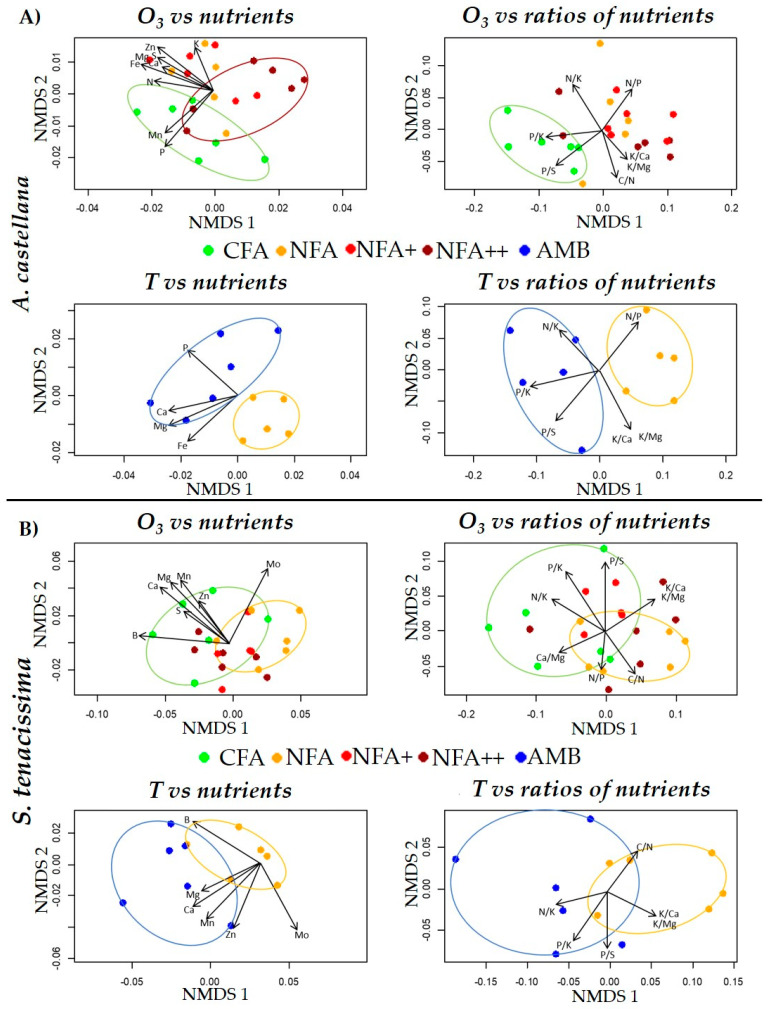
Non-metric multidimensional scaling (NMDS) of nutrients (**left**) and nutrient ratios (**right**) found in *A. castellana* (**A**) and *S. tenacissima* (**B**) in relation to O_3_ and temperature (T) factors. The presence of colored ellipse represents significant differences among treatments following PERMANOVA results. Arrows denote significant correlations of nutrient or nutrient ratios with the NMDS axis.

**Figure 5:**
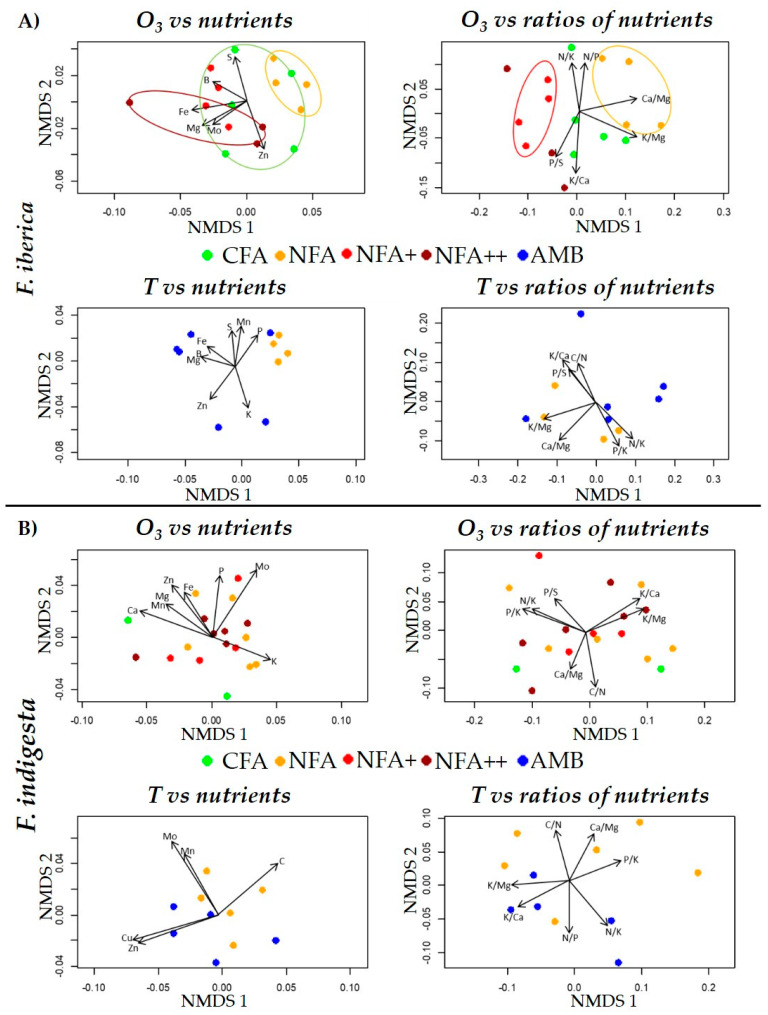
Non-metric multidimensional scaling (NMDS) of nutrients (**left**) and nutrient ratios (**right**) found in *F. iberica* (**A**) and *F. indigesta* (**B**) in relation to ozone and temperature (T) factors. The presence of colored ellipse represents significant differences among treatments following PERMANOVA results. Arrows denote significant correlations of nutrient or nutrient ratios with the NMDS axis.

Nutrients (including C and H content) and nutrient ratios were differentially spatially distributed among the treatments following MNDS (Non-metric multidimensional scaling). MNDS results are presented for *A. castellana* and *S. tenacissima* in Figure 4, and for both *Festuca* especies, *F. indigesta* and *F. iberica*, in Figure 5. The statistical differences among treatments that are shown below are based in PERMANOVA analyses (Table S6). Regarding foliar nutrient content, *A. castellana* plants grown under the lowest O_3_ exposure, the CFA treatment, had more Mn and P than plants grown under the highest O_3_ exposure of the NFA++ (F_3, 19_ = 2.46, *p* = 0.02). In *S. tenacissima*, the concentration of S, Ca, Mg, Mn, B, and Zn was higher in CFA than NFA (F_3, 19_ = 2.34, *p* = 0.03). PERMANOVA analysis also found significant differences among treatments in *F. iberica* (F_3, 12_ = 1.86, *p* = 0.048), with NFA having lower Fe, Mg, and Mo values than CFA. Although NFA+ was also significantly different from NFA, the dispersion of the three NFA+ points calls for caution in the interpretation of these results. However, there were no differences among treatments in *F. indigesta* for foliar nutrient concentration (F_3, 14_ = 0.81, *p* = 0.64).

P/K and P/S nutrient ratios were different in CFA compared with the rest of the O_3_ treatments in *A. castellana* (F_3, 19_ = 3.02, *p* = 0.01). In *S. tenacissima*, only CFA and NFA were marginally different (F_3, 19_ = 2.06, *p* = 0.07) due to different P/S, N/K, P/K, Ca/Mg, C/N, K/Ca, and K/Mg ratios. NFA had lower Ca/Mg and K/Mg ratios than NFA+ in *F. iberica* (F_3, 12_ = 2.8, *p* = 0.01). There were no differences among O_3_ treatments in *F. indigesta* due to nutrient ratios (F_3, 14_ = 0.7, *p* = 0.68).

The lower temperature of AMB compared with NFA resulted in a higher P, Ca, Mg, and Fe content for *A. castellana* (F_1, 9_ = 3.68, *p* = 0.01). AMB had higher amounts of Mg, Ca, Mn, and Zn than NFA (F_1, 10_ = 5.15, *p* = 0.01) in *S. tenacissima*. Temperature caused no difference in nutrients content between AMB and NFA for either *F. iberica* (F_1, 8_ = 2.31, *p* = 0.12) or *F. indigesta* (F_1, 9_ = 1.68, *p* = 0.18). AMB plants presented higher ratios of P/K, P/S, and N/K and lower N/P than NFA plants in *A. castellana* (F_1, 9_ = 5.53, *p* = 0.01). In *S. tenacissima*, AMB was significantly different from NFA due to different ratios of N/K, P/K, K/Ca, and K/Mg (F_1, 10_ = 5.24, *p* = 0.02). There were no differences between AMB and NFA treatments in nutrients ratios in *F. iberica* (F_1, 8_ = 1.68, *p* = 0.21) and *F. indigesta* (F_1, 9_ = 1.57, *p* = 0.23).

### 2.5. Correlations

**Figure 6:**
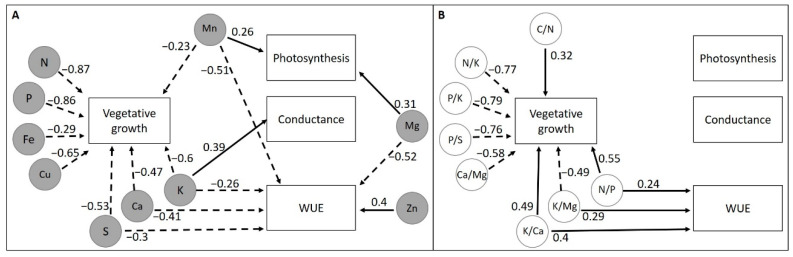
Correlations of (**A**) nutrients and (**B**) nutrient ratios with photosynthesis (**A**), stomatal conductance (g_s_), water use efficiency (WUE), and vegetative growth of the four perennial Mediterranean grasses assayed. Only significant correlations are shown. Solid and dotted arrows denote positive and negative correlations, respectively. Values next to arrows are the correlation values.

Figure 6 summarizes the significant correlations (*p* < 0.05) of nutrients content (Figure 6A) and nutrient ratios (Figure 6B) with gas exchange parameters (A, g_s_, and WUE) and aboveground vegetative biomass (leaf DW) for the four grasses studied. Photosynthetic activity was positively correlated with Mn and Mg, and g_s_ increased with the amount of K. WUE was correlated positively with Zn, N/P, K/Ca, and K/Mg. On the other hand, higher K, Ca, Mg, Mn, and S were correlated with lower WUE values. Leaf DW was positively correlated with C/N, N/P, and K/Ca, but negatively with N, K, Ca, Cu, Fe, Mn, P, S, N/K, P/K, P/S, K/Mg, Ca/Mg.

## 3. Discussion

The present study reproduced the current and foreseen O_3_ levels and temperature increases on the pasture habitats of Mediterranean mountains. Ozone concentrations recorded at a mountain summit of the Spanish Central System yielded AOT40 values, cumulated over a 3-month period, ranging from 13,900 to 19,100 nL L^−1^ h depending among years [12]. These values greatly exceeded the current objectives for plant protection established in the EU directive of air quality (2008/50/EU), and the critical levels of the Air Convention for perennial pastures [9]. The equivalent estimated 3-month AOT40 index for NFA+ and NFA++ treatments in the present assay, comprising a 63-day fumigation period, would be about 12,000 and 22,000 nL L^−1^ h, respectively. Therefore, the O_3_ treatments in the experiment captured the interannual variability of O_3_ in the Iberian mountains. Furthermore, O_3_ concentrations in the NFA++ were also in the range of expected future O_3_ levels in Mediterranean mountain areas by 2050 [24].

Regarding the increase in temperature, previous studies have detected a temperature increment in the Central System of 0.5 °C in the last decade, and projected temperatures in the Mediterranean mountains for 2085 could rise 5 degrees [50,51]. Therefore, the increase in temperature reached in the OTCs with respect to the AMB plots mimicked the expected increase caused by climate warming in the area. Nonetheless, the mean daylight temperatures in this study are within the range of the maximum values recorded in the natural environment of the species tested in the same year of the experiment (25.6 °C; [51,52]. The effect of the temperature increase found in this study could be slightly affected by the small differences between OTC and AMB in the other meteorological factors.

The O_3_ and temperature sensitivity of the Mediterranean perennial grass species tested in this study does not present a single response or pattern. Alternatively, response patterns between species will be discussed in the light of their phylogenetic proximity and their adaptation to drought.

### 3.1. Effects on Plant Growth and Gas Exchange Parameters

As expected, the vegetative growth of Mediterranean perennial grasses showed a tolerant response to O_3_ except in *F. indigesta*. This general lack of response to O_3_ in growth parameters is consistent with previous results with grasses from natural ecosystems. Both Mediterranean annual and temperate perennial grasses have shown none or minor effects on growth under increasing O_3_ exposure, as compared with O_3_ sensitive legumes and forbs [31,33,34,41,42,53]. Despite this fairly widespread O_3_-tolerance of perennial grasses, some species such as *Nardus stricta* responded negatively to the pollutant [54]. In the present study, *F. indigesta* responded to the O_3_ increase following a non-linear pattern: plants developed the highest biomass under the NFA and NFA++ treatments while minimum values were registered in the NFA+ with biomass reductions of 30% compared with CFA. *F. indigesta* showed a greater O_3_-sensitivity than its close phylogenic relative, *F. iberica*, despite the larger growth observed in the latter. *F. iberica* shows preference for wetter soils under natural conditions which would have been favored, as compared with *F. indigesta*, by the growing conditions in this study, where plants were kept with full water availability. A higher growth rate is usually associated with more intense physiological activity, greater gas exchange rates, and O_3_ uptake [9]. Therefore, this study shows that the growth is not a reliable indicator of O_3_ sensitivity for *Festuca* species. Interestingly, the O_3_ levels in the natural mountain habitats of the *Festuca* species at the Spanish Central System during the spring season, when plants do not suffer drought stress and are fully physiologically active [12], are closely reproduced by the NFA+ conditions. Therefore, these results should warn of the current potential risk of O_3_ negative effects in *F. indigesta*-dominated natural habitats, which are widespread in the Spanish Central System [28,55].

Only *A. castellana* responded positively to temperature increases. Considering the absence of soil moisture limitation in the present study, a higher growth under warmer conditions for all species could be expected [56]. During daylight hours, the mean temperature inside the OTCs was 26 °C during the experimental period, which is a common value for the natural habitat of this species in late spring and summer months. However, during the last 10 days of the fumigation experiment, temperatures rose inside the OTCs reaching values up to 40 °C. This late condition could hardly have affected the growth responses, since most of the growth occurred before the heat wave, and plants remained healthy without symptoms of heat stress. Therefore, *A. castellana* could potentially benefit, in the absence of water limitation, from the expected warming in its natural habitat in terms of aboveground biomass growth.

Gas exchange parameters were more sensitive to O_3_ than biomass yield. *F. iberica*, *F. indigesta*, and *A. castellana* were affected differently according to the gas exchange parameter considered, while *S. tenaccissima* showed no effect. Previous studies have found that O_3_ frequently inhibits A as a broad physiological response to the pollutant [57,58,59], response that has also been reported for grass species, and similarly this pollutant can also induce stomatal closure in O_3_-sensitive species [13,22,54,60,61]. In agreement with biomass growth responses, no species showed reductions of A in response to O_3_ except *F. indigesta* and a marginal increase was even recorded in *A. castellana*. Both *Festuca* species showed a similar non-linear response pattern, but this effect was only marginally significant for *F. indigesta.* Strikingly, the highest A values measured in NFA+ were associated with the maximum growth loss in *F. indigesta*. A possible explanation is that photosynthate production under NFA+ was allocated to oxidative damage repair and defence [62,63,64,65], below ground biomass, or reproductive output [60,66]. However, similar A values in the NFA++ treatment would again be allocated to aboveground biomass growth. This kind of hormetic trend has been studied as an O_3_-triggered biphasic response under an oxidative stress [67]. A marginally linear positive response was found in *A. castellana*, not associated with higher aboveground biomass. In this case, like in *F. indigesta*, results suggest that higher amounts of photosyntates may have been allocated to other sinks under increasing O_3_ stress.

The O_3_ tolerance of plant species have been frequently related with stomatal behavior, since higher g_s_ favors O_3_ absorption and greater oxidative damage [68,69,70]. Of the species analyzed here, *S. tenacissima* and *F. iberica* would be the species with the maximum potential g_s_ (based on CFA values). However, both species were tolerant in terms of growth and A. The O_3_-tolerance of the assayed grasses could then be related with a greater plant capacity for detoxification and repair, or a greater allocation to aboveground biomass, which has been considered among the main mechanisms underlying the tolerance of some species [60,66,71].

WUE was the gas exchange parameter most significantly affected by O_3_ exposure in the present study. Ozone can unbalance WUE by inducing changes of different intensity in A and g_s_ [72,73]. Ozone affected WUE in both *Festuca* species, although with different patterns. In *F. iberica*, the pollutant induced a reduction of stomatal opening maintaining A, resulting in a significant increase in WUE with increasing O_3_ exposure. *F. iberica* can be classified as a “water use opportunistic” species [74], based on high g_s_ and low WUE observed under the CFA treatment. However, under scenarios of high O_3_ pollution, *F. iberica* would turn on a more “conservative” water use strategy. Therefore, the physiological response of *F. iberica* to O_3_ levels expected in the future could help to simultaneously limit the effects of drought. Moreover, the WUE of this species was not affected by temperature. Therefore, the results would point towards a good performance of *F. iberica* under future combined scenarios of warming and O_3_ pollution. *F. indigesta* showed the highest WUE under current O_3_ levels in mountain areas (NFA+ treatment) of the species tested in this study, which is in concordance with its better adaptation to low soil water availability growing conditions [44]. However, increasing O_3_ levels might affect the plant adaptation to these growing conditions, as O_3_ induced a quadratic (hormetic) response in WUE, with the lowest WUE found under NFA++. The observed response of WUE to O_3_ in *A. castellana* can also be explained as another hormetic response explained by effects on g_s_: first the rise of O_3_ would induce a stomatal closure and then the excess of oxidative stress by higher O_3_ levels would increase this parameter. This pattern of response could be related with a sluggish response of the stomata under O_3_ stress [13,67,69]. The WUE response of *A. castellana* and *F. indigesta* pointed out in the same direction: both species maintained elevated A values under higher O_3_ exposure, but at the cost of losing efficiency in the gas exchange process. Thus, the projected O_3_ concentrations in mountain areas [24] could limit the tolerance of these species to the expected increases in drought [49] in these areas.

Contrarily to O_3_ effects, the increase in temperature improved WUE rate in *A. castellana* because of the decrease in g_s_ while maintaining A. The higher WUE was in agreement with a greater biomass growth. *S. tenacissima*, an O_3_ tolerant species in terms of biomass growth and gas exchange, was the only species that lowered A values when grown at higher temperatures. *S. tenacissima* was sensitive to warming despite the fact that this species usually inhabits the driest and hottest environments of all the species tested [45,46]. Frequently, under well-watered conditions, a moderate increase in air temperature is accompanied by an increase in A [75,76]. However, the opposite response observed in *S. tennaccissima* could be more related with the hot temperatures reached at the late exposure period which can be affected the functioning of the photosystem II, accordingly with [77] for some herbaceous forbs.

### 3.2. Ozone and Temperature Effects on Foliage Nutrient Content

The sensitivity of foliar nutrient content and nutrient ratios to O_3_ and temperature varied among the species and nutrients, or the nutrient ratios considered, in agreement with the results already published in the literature. *A. castellana* and *S. tenacissima* were the most sensitive species based on the number of affected nutrients (Figures S2 and S3), although O_3_ sensitivity in *S. tenacissima* was based on the small increase in O_3_ from CFA to NFA. *A. castellana* and *S. tenacissima* shared the same P/S, P/K, N/K, Ca, Mg, and Mn responses against the rise of O_3_ and/or temperature. Among *Festuca* species, only *F. iberica* showed some response to O_3_ in terms of nutrient content (Figures S4 and S5). Beyond the species-specific responses, the lack of similarity in the sensitivity of *A.* castellana and *F. iberica* to O_3_ and temperature do not support the hypothesis that species sharing the same habitat would respond similarly to the abiotic factors tested in this study. On the other hand, the lack of effects on nutrient content and ratios in the *Festuca* species suggest that temperature sensitivity is phylogenetically related. Nevertheless, more studies involving more species are needed to unravel whether the nutrient response to O_3_ and temperature is related to habitat adaptation or phylogeny.

Some of the responses found in the present work have been previously described for other species. Subtle effects of O_3_ on the foliar C/N ratio in *S. tenacissima* and the absence of effects in the other species tested, contrast with O_3_-induced effects in foliar N observed in trees, pastures, and crop species, with both increasing and decreasing foliar N trends with O_3_ exposure [16,78,79]. However, this result is in agreement with other studies reporting no effect in foliar N [80,81]. *Beta vulgaris* plants showed reductions in leaf Fe content in response to O_3_ [18], like in *F. iberica*. Again, the O_3_ effect on *S. tenacissima* promoting an increase in K/Ca, was also observed in the stomatal guard cells of the O_3_-injured leaves of *Betula pendula* [82]. Ozone induced decreases of foliar Mg and changes in the K/Mg ratio in *F. iberica* and *S. tenacissima* have also been described for *Solanum tuberosum* and *Beta vulgaris* [18,19,83]. Other studies, however, do not show O_3_ effects on foliar nutrient concentration in species such as *Lolium perenne* or *Trifolium repens*, or a mixture of *Trifolium pratense*, *Phleum pratense*, and *Festuca pratensis* [80,84] Temperature effects on foliar P and Fe in *A. castellana* have also been described in *Coffea canephora* and in plants from a dwarf shrub-dominated ecosystem [85,86]. However, the negative temperature effect on Mg in *A. castellana* was not found in Mediterranean scrub [87]. These results show that O_3_ and temperature effects on foliar nutrient content and ratios seem to be species-specific and variable between nutrients. More studies would be needed for understanding general response patterns.

Nutrient and nutrient ratios were more correlated with vegetative growth than with gas exchange parameters. The influence of O_3_ and temperature on leaf nutrient concentration and the relationship with vegetative growth, A, g_s_, and WUE constitute a complex web of interactions (Figures S2–S5). Considering all four species together, the vegetative growth was negatively correlated with most of the macro- and micronutrients and the different ratios considered (Figure 6). The dilution of nutrient content related with biomass growth has been shown for N, P, and K [88,89]. These nutrients are directly or indirectly related to plant development [90,91]. In general, N- and P-deficiency inhibits leaf growth and Fe is mostly located in the chloroplast of growing leaves [90,91]. In *A. castellana*, the temperature effect on leaf nutrients could be explained by a dilution effect due to increases in vegetative growth under the warmest treatment. The N/P ratio was also positively correlated with vegetative growth in this species, which is in agreement with temperature-induced increases in this ratio. None of effects of O_3_ on foliar nutrient content could be related with effects on biomass growth in any of the four species. However, O_3_ effects on foliar nutrient contents in *A. castellana*, *F. iberica*, or *S. tenacissima* found in this short-term exposure study may indicate that effects on plant growth could appear under long-term high O_3_ exposures.

In the present study, Mn and Mg were positively correlated with A. In fact, Mn and Mg act synergistically in the basal metabolism of electron transport reactions in photosynthesis [92,93]. An ozone-induced decrease in foliar Mg has been related with chlorophyll loss and A decline [18,19,83]. In contrast to this, O_3_ exposure increased A in *A. castellana* associated with a decrease in leaf Mn content (Figure S2). However, in *S. tenacissima* the decrease in Mn and Mg under the high temperature treatment could be associated with the decrease in A (Figure S2). Foliar K was positively correlated with g_s_ across species. This correlation is in accordance with the stomatal hydration regulatory function of K that produce the stomata opening due to increased turgor in guard cells [90,92]. Despite O_3_ and temperature effects found on the g_s_ of *A. castellana* and *F. iberica*, leaf K content was not affected by the experimental treatments in any of the four species studied (Figures S2 and S5). Previous studies neither found significant effects on the K-levels of potato or wheat exposed to increased levels of O_3_ [19,94]. S, Ca, K, Mg, and Mn foliar concentrations were negatively correlated with WUE while Zn and N/P, K/Ca, and Ca/Mg ratios were positively correlated (Figure 6). These relationships can be explained through the importance of some of these elements on A or g_s_ processes. Stomatal opening by K accumulation is promoted by Ca signaling [90,91,92] which could result in decreased WUE. Zn-mediated stability of membrane integrity [91,93] may explain the higher WUE under high foliar Zn levels while decreases in foliar Mg could reduce WUE via reductions in A. The O_3_-induced decreases in Mn and P and in the P/K and P/S ratios in *A. castellana* can be associated directly or indirectly with the observed quadratic effect on WUE. However, the quadratic effect of O_3_ on WUE in *F. indigesta* was not associated with any significant change in foliar nutrient concentration. With respect to the temperature effects in *A. castellana*, the increase observed in WUE was associated with decreases in Ca and Mg concentration and a reduction in the N/P ratio.

## 4. Material and Methods

### 4.1. OTC Experiment

Plant exposure to O_3_ treatment levels was performed in an NCLAN-type OTC facility (adapted from the original design by [95]) located in central Spain (450 m.a.s.l., 40°3′ N, 4°26′ W). Plants were exposed to four O_3_ treatments: charcoal-filtered air (CFA), non-filtered air (NFA) reproducing ambient levels, non-filtered air supplemented with 20 nL L^−1^ of O_3_ (NFA+), and non-filtered air supplemented with 40 nL L^−1^ of O_3_ (NFA++). Each O_3_ treatment was replicated 3 times in 12 equally built OTCs randomly distributed in 3 lines (blocks) avoiding shading effects between OTCs. One chamber-less plot (AMB) per block was considered to control the chamber effect and to study the consequences of temperature increase on species.

Ozone was produced from pure O_2_ through an O_3_ generator (A2Z Ozone, Inc., Louisville, KY, USA) and supplied to the NFA+ and NFA++ plots 8 h day^−1^ (7:00 to 15:00 GTM) and 7 days week^−1^. Ozone concentrations inside each chamber and AMB plots were monitored continuously above plant canopy using a UV-absorbance O_3_ monitor (ML^®^ 9810B, Teledyne, Thousand Oaks, CA, USA) with an automated time-sharing system sampling all the OTCs and AMB plots of each line sequentially. Another O_3_ monitor within the fumigation system registered continually ambient O_3_ levels to contrast with the NFA and AMB treatments, as a double check of the accuracy of O_3_ values. Both O_3_ monitors were calibrated at the start of the fumigation treatments following the recommended company protocols. More information about the facility can be found in [22]. Figure S1 shows a picture of the OTCs and AMB plots.

The AOT40 index (nL L^−1^ h) was used to describe the O_3_ exposure. This index was calculated as the accumulated hourly O_3_ concentration over the threshold of 40 nL·L^−1^ during daylight (PAR > 100 μmol·m^−2^ s^−1^) hours through the O_3_-fumigation period [96]. The 8h-mean O_3_ concentration (nL·L^−1^) from 7:00–15:00 h GMT was also considered. Air temperature (T, °C), air relative humidity (RH, %), and photosynthetic active radiation (PAR, μmol·m^−2^·s^−1^) inside one OTC were continuously measured above canopy using AM2315 (T and RH; Adafruit Industries LLC, New York, NY, USA) and Apogee SQ 110 (PAR; Apogee Instruments, Inc., Logan, UT, USA) sensors. Ambient T, RH, and PAR were continuously measured using STH-5031 (T and RH; GEONICA, S.A., Madrid, Spain) and LI-200SZ (PAR; GEONICA, S.A., Madrid, Spain) sensors situated in the meteorological station of the OTC facility.

### 4.2. Plant Material

Established seedlings of *A. castellana*, *S. tenacissima*, *F. indigesta*, and *F. iberica* grown from seeds collected from natural populations of Spanish Central System area were transplanted to 2 L pots during 2017 spring. A mix of peat, vermiculite, and perlite (60:20:20) was used as plant substrate. The total number of plants varied among species, with 90, 88, 77, and 51 for *A. castellana*, *S. tenacissima*, *F. iberica*, and *F. indigesta*, respectively. Plants were kept outdoors since transplantation. They were frequently irrigated and fertilized to meet plant demand, until the start of the experiment. On 15 April 2018, 3 days prior to the start of the O_3_-exposure, plants were cut to 5 cm from the substrate. These species, adapted to cattle browsing, present a good regrowth after biomass cut. On 18 April, plants were randomly allocated to OTCs or AMB plots to start the O_3_ fumigation experiment. The 4 species were exposed to the same O_3_-treatments that lasted 68, 63, 62, and 57 days after the start of the exposure (DaS) for *A. castellana*, *S. tenacissima*, *F. indigesta*, and *F. iberica*, respectively.

### 4.3. Vegetative Growth

At the end of the O_3_-fumigation period, plants were cut again to 5 cm from the substrate to obtain the final aboveground biomass. Samples were dried at 60 °C until constant weight. The O_3_ effect on the dry weight biomass (biomass DW, g) was considered to discuss the O_3_-effect on the vegetative growth of the species. The number of samples per treatment and species are shown in Table S1.

### 4.4. Gas Exchange

Leaf-level net photosynthesis (μmol CO_2_ m^−2^s^−1^) and stomatal conductance to water vapor (mol H_2_O m^−2^s^−1^) were measured using a portable LICOR-6400 infra-red gas analysis system (LiCor Inc., Lincoln, NE, USA). The Water Use Efficiency (WUE; μmol CO_2_ mol^−1^ H_2_O) was calculated from the A/g_s_ ratio. In order to increase the leaf area for the gas exchange measurements, a group of intact leaves was introduced in the LICOR chamber. Each individual measurement was corrected per projected leaf area based on the scanned image of leaf sections measured with ImageJ software [97]. The number of samples per treatment and species are shown in the Table S1. Gas exchange measurements were evenly distributed between 9 and 13 h (GMT) among all treatments, from 28 to 35 days after the start of the fumigation date depending on the phenological stage of each species (Table S2). Air humidity and temperature during the measurements ranged 36–49% and 20–25 °C, respectively, and PAR was maintained at 1000 μmol m^−2^ s^−1^, allowing maximum g_s_ according to previous measurements (Table S2). One measurement per plant was taken on the representative biomass of the plant, avoiding the youngest leaves in growth and the oldest senescent leaves. The arrival of a heat wave towards the end of the ozone fumigation experiment prevented the measurement of gas exchange on ambient *F. indigesta* plants.

### 4.5. Macro- and Micronutrient Composition

Total aerial biomass DW from the different plants was individually milled and pooled to obtain a minimum of 2 independent samples (no plant was repeated in the mixtures) per OTC and AMB plot for elemental content analysis. The number of samples per treatment and species are shown in Table S1. Total carbon and hydrogen content (C and H), macronutrients (N, K, Ca, Mg, P, and S) and micro-nutrients (B, Cu, Fe, Mn, Mo, and Zn) were analyzed and the ratios C/N, N/K, N/P, P/K, P/S, K/Ca, K/Mg, and Ca/Mg were also calculated. The total content of C, H, and N were determined by combustion using an elemental analyzer. Samples were digested under controlled conditions for putting quantitatively into solution these macronutrients (K, Ca, Mg, P, and S) and micronutrients (B, Cu, Fe, Mn, Mo, and Zn). Concentrations of these elements were determined by inductively coupled plasma optical emission spectrometry (ICP-OES). More details on sample preparation and elemental analyses are provided in the suplementary information.

### 4.6. Statistical Analysis

To analyze the effect of O_3_ and temperature factors separately on vegetative growth, A, g_s_, and WUE, two sets of linear mixed models (LMM) were performed. The first set of LMM considered the O_3_ treatments (CFA, NFA, NFA+, and NFA++). In the second set, the comparison between AMB and NFA was considered to study the effect of temperature. For each species, the individual plants were the units of replication. Moreover, to control the possible influence of the experimental design in plants growth, A, g_s_, and WUE responses to the fixed factors (O_3_ and temperature), block (line), and O_3_ treatment nested within line were considered as random factors. For each analysis, the AIC (Akaike information criterion) value was used to choose the most parsimonious option between the models including only line as a random factor or the nested model. Linear O_3_ effects were evaluated using linear a priori contrasts to test the stated hypothesis. Moreover, quadratic and cubic responses were also tested. A priori contrasts in linear mixed models were based on [98]. Linear mixed models were performed using lme function (nlme package; [99]). To test the hypotheses of a linear, quadratic, or cubic effect the contr.poly function [100] was used. The model with the lowest *p*-value was chosen among lineal, quadratic, and cubic options for each analysis. Temperature effects were tested using ANOVA function.

To evaluate the O_3_ and temperature effect on leaf nutrient composition, PERMANOVA and Non-Metric Multidimensional Scaling (NMDS) analyses were performed using leaf nutrient concentration and nutrient ratios as a multivariate trait among treatments. To prevent PERMANOVA from being largely influenced by the high levels of some elements, the data were transformed using the square root, and a similarity matrix was made by Bray–Curtis approximation [101]. A total of 9999 permutations were chosen, and PERMANOVA was performed using vegan package (Oksanen et al., 2020). Pairwise analyses were performed when PERMANOVA found differences among O_3_-treatment using pairwise.adonis2 function (package pairwiseAdonis; [102]). Two-dimension NMDS were used to represent the elemental composition multidimensional data. Function metaMDS was used to perform the NMDS (vegan library; [103]). Correlations of nutrients and nutrient ratios with vegetative growth, A, g_s_, and WUE were also performed using vegan package [103]. All the statistical analyses described were carried out in R software [100]. Significant statistical differences were considered at *p*-values lower than 0.05. Marginally statistical differences were considered at *p*-values from 0.1 to 0.05.

## 5. Conclusions

Gas exchange parameters, in particular WUE, and leaf nutrient concentration were more sensitive to O_3_ and temperature increases than aboveground biomass growth in the Mediterranean perennial grass species assayed in this experiment.

The O_3_ sensitivity classification of grasses based on growth and gas exchange does not match with that based on foliar nutrients. *S. tenaccissima* was the most tolerant to O_3_ in terms of growth and gas exchange. However, *A. castellana* were the most sensitive to the pollutant in terms of nutrients. In both *Festucas*, gas exchange, growth, and leaf nutrient content had different responses to O_3_. The O_3_-effects on foliar nutrients and their ratios, although substantial, did not have an early impact on plant growth. Longer term exposures will be needed to understand the potential consequences of these changes for the growth and survival of these species.

*S. tenacissima* and *A. castellana* were the two most sensitive grasses to temperature increase. *S. tenacissima* was the only one that decreased A at high temperatures, despite the fact that it usually inhabits the driest and hottest environments of all the species tested. *A. castellana* was the only species that responded positively in terms of aboveground biomass and WUE when grown in a warmer environment. This species could benefit from climate warming when water is not a limiting factor. However, it was the most temperature-sensitive in terms of foliar nutrients, so the long-term effects may differ from the ones found in this experiment. Only leaf nutrient content responses to temperature seem to be phylogenetically constrained in *Festuca* species.

The results showed that responses to O_3_ and temperature can have different species-specific effects on plant physiology, potentially altering the ability of plants to cope with environmental stresses and changing the relationships among species that share the same habitat. Therefore, climate warming and O_3_ pollution should be considered as two important threats to Mediterranean perennial pastures in the framework of the Global Change. More experimental work would be needed to better understand the behaviour of these complex Mediterranean pastures in response to combinations of stress factors.

## Figures and Tables

**Table 1:** Ozone exposure during the whole experimental period for the different grasses: accumulated O_3_ exposure index AOT40 and O_3_ 8h-mean. DaS, days after the start of the O_3_ exposure.

	AOT40 (nL L^−1^ h)						O_3_ 8h-Mean (nL L^−1^)				
	DaS	AMB	CFA	NFA	NFA+	NFA++	AMB	CFA	NFA	NFA+	NFA++
*S. tenacissima*	68	5780	189	4285	9340	16,677	43.4	21.1	40.7	50.8	63.3
*A. castellana*	63	4622	187	3399	8466	15,807	42.3	20.9	39.8	50.7	64.1
*F. iberica*	62	4456	187	3286	8349	15,692	42.2	20.8	39.7	50.8	64.4
*F. indigesta*	57	3964	187	2951	8072	15,321	41.9	20.9	39.8	51.6	66.2
Mean	63	4705	187	3480	8557	15,875	42.5	20.9	40.0	51.0	64.5

**Table 2:** Mean meteorological conditions during the experimental assay for the 8-h daylight period 7:00–15:00 (GMT) inside the OTCs and in the AMB plots. Mean and maximum air temperature (mean T, max T), air relative humidity (RH), photosynthetic active radiation (PAR) and vapor pressure deficit (VPD, kPa).

	Mean T (°C)	Max T (°C)	RH (%)	PAR (µmol m^−2^ s^−1^)	VDP (kPa)
AMB	20.4	37.2	49.4	1149	1.21
OTCs	25.7	43.2	47.4	991	1.74

## Data Availability

Publicly available datasets were analyzed in this study. This data can be found here: http://rdgroups.ciemat.es/web/geca-ciemat/ (accessed on 20 January 2023).

## Supplementary Materials

The following supporting information can be downloaded at: https://www.mdpi.com/article/10.3390/plants12030664/s1, Figure S1: Picture of two OTCs and one AMB plot of the OTC facility; Description of the description of the total content of C, H, macro- and micro-nuctrient determitaion; Table S1: Number of samples per treatment and specie for Vegetative growth (dry weight), Gas Exchange (A, g_s_ and WUE) and Nutrient (contents and rates) measurements; Table S2. LICOR measurements conditions for photosynthesis and conductance; Table S3: A priori contrast results of the lineal effect, quadratic effect and cubic effect for each specie in vegetative growth, A, g_s_ and WUE; Table S4: Temperature effect results on vegetative growth, A, g_s_ and WUE; Table S5: The structure of the random part of the model for each trait (vegetative growth, photosynthesis, conductance and WUE) and species; Table S6: PERMANOVA tables of nutrient and nutrients ratios for O_3_ and temperature; Figure S2: Ozone and temperature observed effects on growth and gas exchange measured traits and nutrients in *A. castellana*; Figure S3: Ozone and temperature observed effects on growth and gas exchange measured traits and nutrients in *S. tenacissima*; Figure S4: Ozone and temperature observed effects on growth and gas exchange measured traits and nutrients in *F. indigesta;* Figure S5: Ozone and temperature observed effects on growth and gas exchange measured traits and nutrients in *F. iberica.*
