# Supplementary material for: Ozone and Temperature May Hinder Adaptive Capacity of Mediterranean Perennial Grasses to Future Global Change Scenarios
Source: Plants (Basel). 2023 Feb 2;12(3):664. doi: 10.3390/plants12030664 (PMC9920155; doi:10.3390/plants12030664)

Suplementary information

Figure S1. Picture of two OTCs and one AMB plot of the OTC facility.

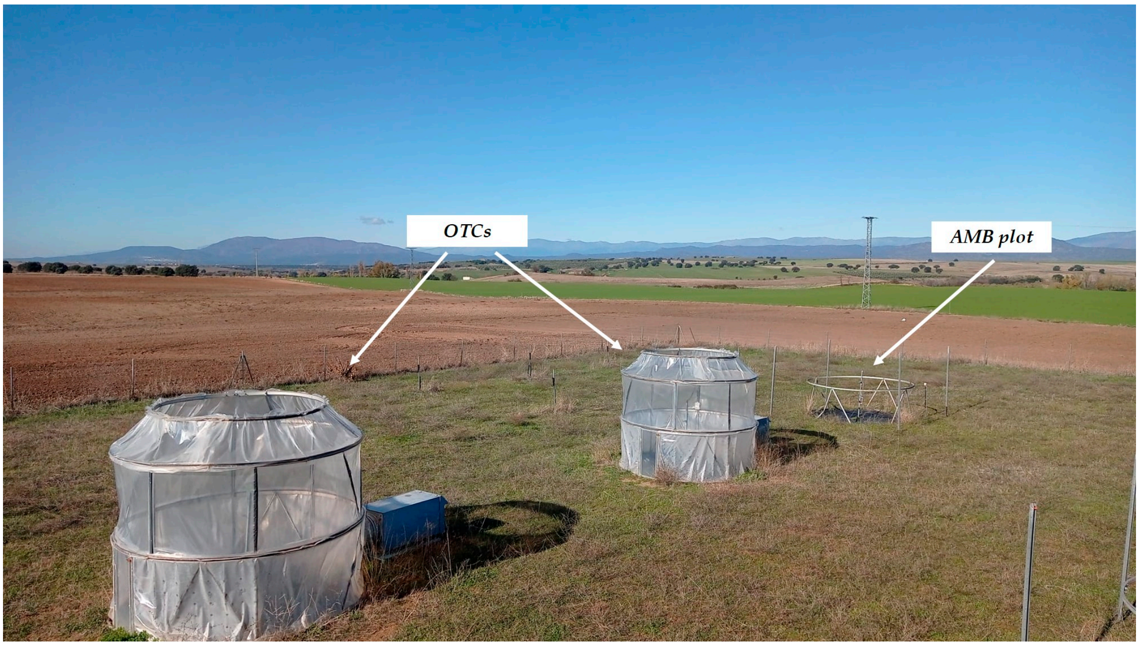

Description of the description of the total content of C, H, macro- and micro-nuctrient determitaion:

Samples were analyzed in the CIEMAT lab of the Chemistry Division. The total content of carbon, hydrogen and nitrogen were determined by combustion employing a LECO TruSpec CHN elemental analyzer (LECO INSTRUMENTOS S.L., Madrid, Spain). C, N and H contents were determined by heating to temperature of at least 900ºC in the presence of oxygen gas. Mineral and organic compounds were oxidized and/or volatilized to carbon dioxide (CO_2), water (H_2O), nitrogen oxides (NO_x) and molecular nitrogen (N_2). The amount of carbon dioxide and water was measured by an infrared detection method. In order to quantify macro-nutrients (K, Ca, Mg, P, and S) and micro-nutrients (B, Cu, Fe, Mn, Mo, and Zn), previous treatments of the samples are required for putting quantitatively into solution these elements. Plant samples were digested in a DigiPrep block (SCP Science, Quebec, Canada) equipped with a temperature–time programmable controller by using open polypropylene tubes with reflux caps in a graphite heating block at high temperature. Briefly, 250 mg dry weight plant samples were mixed with 6 ml of nitric acid (HNO_3) (67-69 %) and 2 ml of hydrofluoric acid (HF) (47-51 %). They were subjected to heat reaching 120°C in 40 min and being held for 60 min, and then cooled, filtered, diluted to the mark (50 ml) with ultrapure water and preserved for subsequent analysis. Concentrations of each macro- and micro-nutrient were determined by inductively coupled plasma optical emission spectrometry (ICP-OES) using a benchtop dual view ICP-OES with a vertical torch, Agilent 5900 SVDV model (Agilent Technologies Spain, S.L., Spain) equipped with an Advanced Valve System (AVS 7) and with the optic system purged with argon. Multielement standard solutions for calibration were daily prepared by successive dilutions of macro- and micro- nutrients at different concentrations. Plant simple digestions

were analyzed directly or diluted when the concentration are higher than those of the most concentrated standard. For theses analyses all reagents were purchased from Merck KGaA (Darmstadt, Germany). The reagents were ultrapure analysis quality. Ultrapure water (resistivity ≥18.2 MΩ cm) was obtained by a Milli-Q Element A10 (Millipore, Bedford, MA, USA). Stock standard solutions 1000 mg l^{-1} or each single element in 2% HNO_3 were Spex CertiPrep (Fisher Scientific SL, Madrid, Spain) were used to prepare the corresponding multielement stock standard solutions by successive dilutions. Argon gas of purity higher than 99.999% was used in the ICP-OES instrument (Air Liquide, Spain).

Table S1. Number of samples per treatment and specie for Vegetative growth (dry weight), Gas Exchange (A, g_s and WUE) and Nutrient (contents and rates) measurements.

Measurement	Treatment	*A. castellana*	*S. tenacissima*	*F. indigesta*	*F. iberica*
	CFA	18	17	6	16
	NFA	18	18	11	15
**Dry weight**	NFA+	18	17	11	15
	NFA++	18	18	12	13
	AMB	18	18	11	18
total per specie		90	88	51	77
	CFA	6	9	4	8
	NFA	7	9	6	7
**Gas exchange**	NFA+	7	9	6	8
	NFA++	6	9	6	9
	AMB	6	9	-	4
total per specie		32	45	22	36
	CFA	6	6	2	5
	NFA	5	6	6	4
**Nutrients**	NFA+	6	5	4	4
	NFA++	6	6	6	3
	AMB	6	6	5	6
total per specie		29	29	23	22

Table S2. LICOR measurements conditions for photosynthesis and conductance. Days of O_3 exposition (Days), temperature (Tª), relative humidity (RH) and photosynthetic active radiation (PAR).

	Days	RH (%)	Tª (Cº)	PAR µmol (m^{-2} s^{-1})
S. tenacissima	35	49	20	1000
A. castellana	33	48	20	1000
F. iberica	34	36	22	1000
F. indigesta	28	42	25	1000

Table S3. A priori contrast results of the lineal effect, quadratic effect and cubic effect for each specie in vegetative growth, A, g_s and WUE.

trait	specie	a priori contrast	Df	Std. error	t	P
vegetative growth	*A. castellana*	intercept	66	0.43	43.34	
		lineal effect	66	0.86	0.21	0.83
		quadratic effect	66	0.86	-1.18	0.24
		cubic effect	66	0.86	0.39	0.69
	S. tenacissima	intercept	64	0.95	12.91	0
		lineal effect	64	0.6	1.12	0.27
		quadratic effect	64	0.6	1.05	0.3
		cubic effect	64	0.6	0.95	0.35
	F. indigesta	intercept	28	0.48	6.65	0
		lineal effect	6	0.99	-0.21	0.84
		quadratic effect	6	0.96	-0.39	0.71
		cubic effect	6	0.94	2.22	0.07
	F. iberica	intercept	46	0.83	5.42	0
		lineal effect	6	1.4	0.98	0.96
		quadratic effect	6	1.41	0.37	0.48
		cubic effect	6	1.42	0.26	0.53
photosyntesis	*A. castellana*	intercept	20	1.69	16.65	0
		lineal effect	20	2.36	1.93	0.07
		quadratic effect	20	2.3	0.47	0.65
		cubic effect	20	2.21	0.03	0.97
	S. tenacissima	intercept	30	1	32.43	0
		lineal effect	30	2	-0.27	0.79
		quadratic effect	30	2	1.61	0.12
		cubic effect	30	2	0.27	0.79
	F. indigesta	intercept	16	4.12	6.11	0
		lineal effect	16	3.09	0.5	0.63
		quadratic effect	16	2.96	0.38	0.71
		cubic effect	16	2.82	-2.03	0.06
	F. iberica	intercept	26	1.53	21.67	0
		lineal effect	26	2.98	-0.07	0.94
		quadratic effect	26	3.05	0.016	0.88
		cubic effect	26	3.13	-1.29	0.21

Table S3 (continued). A priori contrast results of the lineal effect, quadratic effect and cubic effect for each specie in vegetative growth, A, g_s and WUE.

trait	specie	a priori contrast	Df	Std. error	t	P
conductance	*A. castellana*	intercept	20	0.045	10.99	0
		lineal effect	20	0.049	0.94	0.36
		quadratic effect	20	0.048	1.79	0.09
		cubic effect	20	0.046	-0.18	0.86
	S. tenacissima	intercept	30	0.06	13.77	0
		lineal effect	30	0.09	-0.71	0.48
		quadratic effect	30	0.09	1.42	0.17
		cubic effect	30	0.09	0.72	0.18
	F. indigesta	intercept	16	0.06	6.14	0
		lineal effect	16	0.06	0.69	0.5
		quadratic effect	16	0.06	1.52	0.15
		cubic effect	16	0.06	-1.14	0.27
	F. iberica	intercept	26	0.06	13.94	0
		lineal effect	26	0.12	-1.21	0.24
		quadratic effect	26	0.12	-0.05	0.96
		cubic effect	26	0.12	-1.75	0.09
WUE	*A. castellana*	intercept	20	2.13	27.4	0
		lineal effect	20	3.33	1.09	0.29
		quadratic effect	20	3.25	-2.22	0.04
		cubic effect	20	3.13	0.22	0.82
	S. tenacissima	intercept	30	0.06	61.98	0
		lineal effect	30	0.08	0.47	0.64
		quadratic effect	30	0.08	-0.67	0.51
		cubic effect	30	0.08	-0.97	0.34
	F. indigesta	intercept	16	2.71	26.37	0
		lineal effect	16	5.66	-0.64	0.53
		quadratic effect	16	5.42	-2.37	0.03
		cubic effect	16	5.18	-0.28	0.79
	F. iberica	intercept	26	2	21.71	0
		lineal effect	26	3.9	2.26	0.03
		quadratic effect	26	4	0.58	0.57
		cubic effect	26	4.1	2.08	0.05

Table S4. Temperature effect results on vegetative growth, A, g_s and WUE.

trait	specie		num Df	den Df	F	P
vegetative growth	*A. castellana*	intercept	1	32	737.53	<0.0001
		ozone	1	32	3.95	0.055
	S. tenacissima	intercept	1	32	493.72	<0.0001
		ozone	1	32	0.1	0.76
	F. indigesta	intercept	1	16	26.99	0.0001
		ozone	1	2	0.11	0.77
	F. iberica	intercept	1	26	33.48	<0.0001
		ozone	1	2	0.01	0.94
photosyntesis	*A. castellana*	intercept	1	9	60.18	0
		ozone	1	9	0.53	0.48
	S. tenacissima	intercept	1	14	1384.7	<0.0001
		ozone	1	14	7.24	0.02
	F. iberica	intercept	1	7	57.26	0.0001
		ozone	1	7	1.73	0.23
conductance	*A. castellana*	intercept	1	9	44.58	0.0001
		ozone	1	9	3.59	0.09
	S. tenacissima	intercept	1	14	203.4	<0.0001
		ozone	1	14	0.17	0.69
	F. iberica	intercept	1	7	109.5	<0.0001
		ozone	1	7	1.75	0.23
WUE	*A. castellana*	intercept	1	9	782.18	<0.0001
		ozone	1	9	5.65	0.04
	S. tenacissima	intercept	1	14	4114.3	<0.0001
		ozone	1	14	0.49	0.49
	F. iberica	intercept	1	7	256.5	<0.0001
		ozone	1	7	0.75	0.41

Table S5. The structure of the random part of the model for each trait (vegetative growth, photosynthesis, conductance and WUE) and species. Line is the fumigation line of the OTC facility and nested is the O_3 treatment nested within line.

**Specie**	**Trait**	**Fixed factor**	**Random factors**
A. castellana	Vegetative growth	O_3	line
S. tenacissima	Vegetative growth	O_3	line
F. iberica	Vegetative growth	O_3	nested
F. indegesta	Vegetative growth	O_3	nested
A. castellana	Vegetative growth	temperature	line
S. tenacissima	Vegetative growth	temperature	line

F. iberica	Vegetative growth	temperature	nested
F. indigesta	Vegetative growth	temperature	nested
A. castellana	Photosyntesis	O_3	line
S. tenacissima	Photosyntesis	O_3	line
F. iberica	Photosyntesis	O_3	line
F. indegesta	Photosyntesis	O_3	line
A. castellana	Photosyntesis	temperature	line
S. tenacissima	Photosyntesis	temperature	line
F. iberica	Photosyntesis	temperature	line
A. castellana	Conductance	O_3	line
S. tenacissima	Conductance	O_3	line
F. iberica	Conductance	O_3	line
F. indegesta	Conductance	O_3	line
A. castellana	Conductance	temperature	line
S. tenacissima	Conductance	temperature	line
F. iberica	Conductance	temperature	line
A. castellana	WUE	O_3	line
S. tenacissima	WUE	O_3	line
F. iberica	WUE	O_3	line
F. indegesta	WUE	O_3	line
A. castellana	WUE	temperature	line
S. tenacissima	WUE	temperature	line
F. iberica	WUE	temperature	line

Table S6. PERMANOVA tables of nutrient and nutrients ratios for O_3 and temperature.

Specie	multivariable	PERMANOVA Table	Df	SumOfSqs	F	P
A. castellana	nutrients	Ozone treatments	3	0.0004	2.46	0.02
		residuals	19	0.001		
	nutrient ratios	Ozone treatments	3	0.004	3.02	0.001
		residuals	19	0.008		
	nutrients	temperature	1	0.0002	3.68	0.01
		residuals	9	0.0006		
	nutrient ratios	temperature	1	0.0004	5.53	0.01
		residuals	9	0.001		
S. tenacissima	nutrients	Ozone treatments	3	0.001	2.34	0.03
		residuals	19	0.002		
	nutrient ratios	Ozone treatments	3	0.005	2.06	0.07
		residuals	19	0.02		
	nutrients	temperature	1	0.001	5.15	0.01
		residuals	10	0.001		
	nutrient ratios	temperature	1	0.004	5.24	0.02
		residuals	10	0.008		
F. indigesta	nutrients	Ozone treatments	3	0.003	0.81	0.64
		residuals	14	0.02		
	nutrient ratios	Ozone treatments	3	0.003	0.7	0.68
		residuals	14	0.02		
	nutrients	temperature	1	0.002	1.68	0.18
		residuals	9	0.01		
	nutrient ratios	temperature	1	0.002	1.57	0.23
		residuals	9	0.01		
F. iberica	nutrients	Ozone treatments	3	0.001	1.86	0.048
		residuals	12	0.02		
	nutrient ratios	Ozone treatments	3	0.01	2.8	0.01
		residuals	12	0.02		
	nutrients	temperature	1	0.0003	2.3	0.12
		residuals	8	0.001		
	nutrient ratios	temperature	1	0.003	1.68	0.21
		residuals	8	0.02		

Figure S2. Ozone and temperature observed effects on growth and gas exchange measured traits and nutrients in *A. castellana*. Upper box, effects on nutrients and nutrients rates leaf amounts: red and yellow arrows denote the effects on nutrients of ozone (O_3) and temperature (T), respectively; pink, blue and green denote the correlation of nutrients (grey circles) and nutrient rates (white circles) with the traits; solid arrows indicate positive relationships and dashed arrows indicate negative relationships. Grey box, observed effect on traits: red and yellow arrows denote the effects on nutrients of O_3 and T; straight and solid arrows indicate positive relationships and straight dashed arrows indicate negative relationships; curve solid and dashed arrows mark concave and convex trend, respectively.

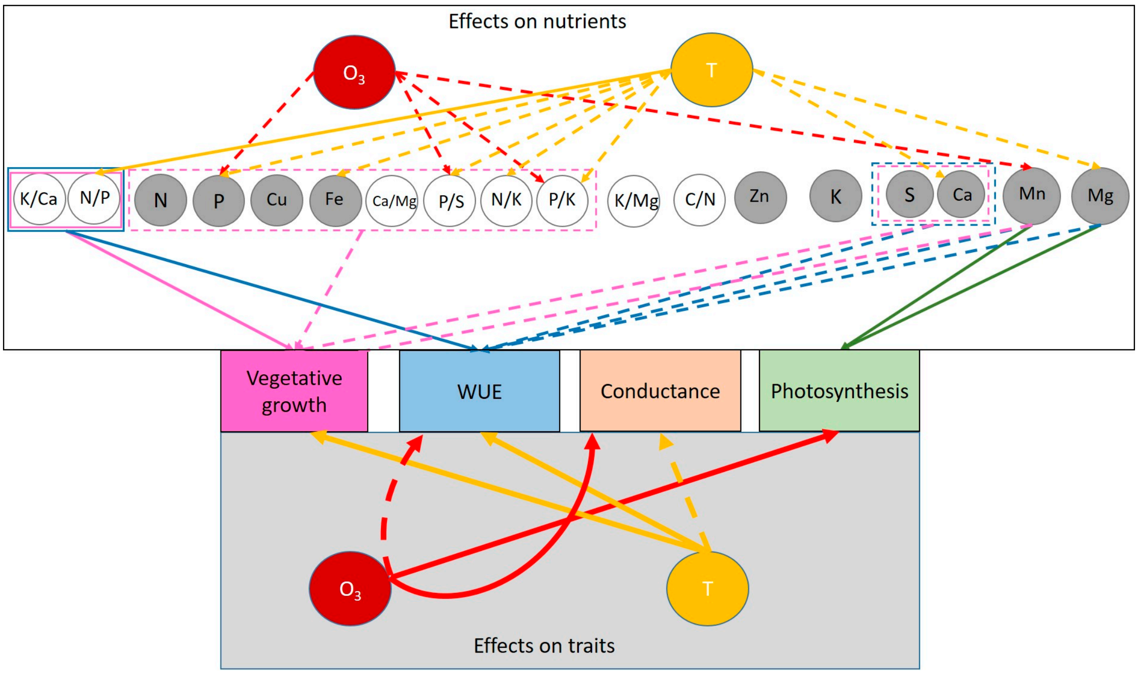

Figure S3. Ozone and temperature observed effects on growth and gas exchange measured traits and nutrients in *S. tenacissima*. Upper box, effects on nutrients and nutrients rates leaf amounts: red and yellow arrows denote the effects on nutrients of ozone (O_3) and temperature (T), respectively; pink, blue and green denote the correlation of nutrients (grey circles) and nutrient rates (white circles) with the traits; solid arrows indicate positive relationships and dashed arrows indicate negative relationships. Grey box, observed effect on traits: red and yellow arrows denote the effects on nutrients of O_3 and T; straight and solid arrows indicate positive relationships and straight dashed arrows indicate negative relationships; curve solid and dashed arrows mark concave and convex trend, respectively.

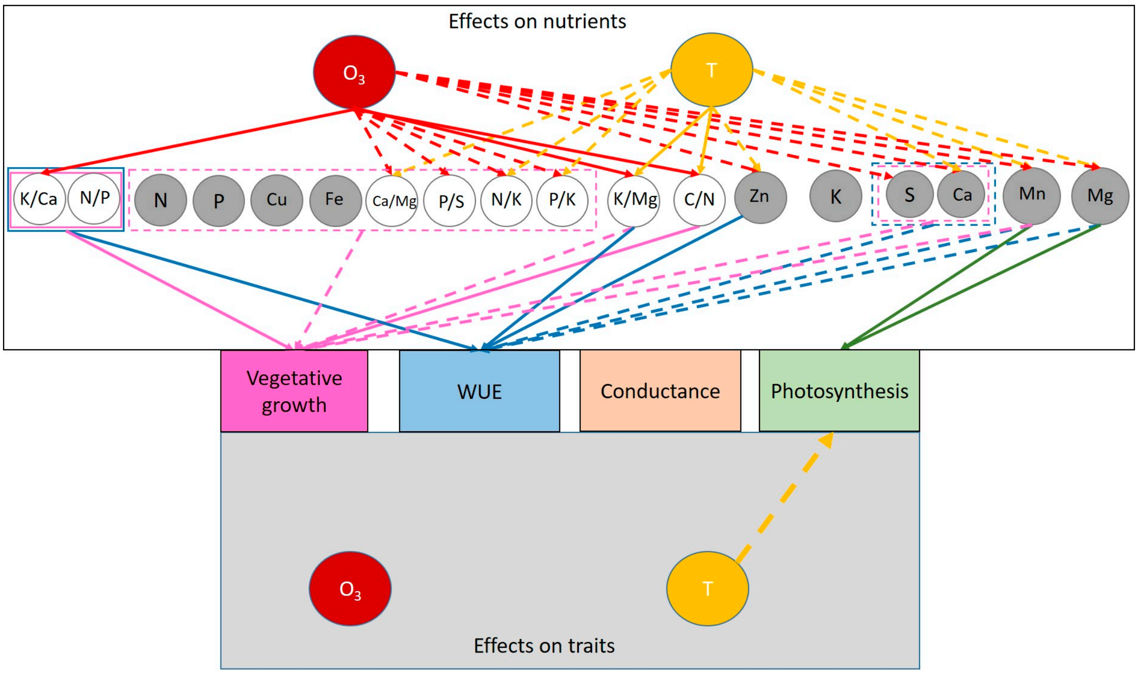

Figure S4. Ozone and temperature observed effects on growth and gas exchange measured traits and nutrients in *F. indigesta*. Upper box, effects on nutrients and nutrients rates leaf amounts. Grey box, observed effect on traits: red and yellow arrows denote the effects on nutrients of O_3 and T; straight and solid arrows indicate positive relationships and straight dashed arrows indicate negative relationships; curve solid and dashed arrows mark concave and convex trend, respectively.

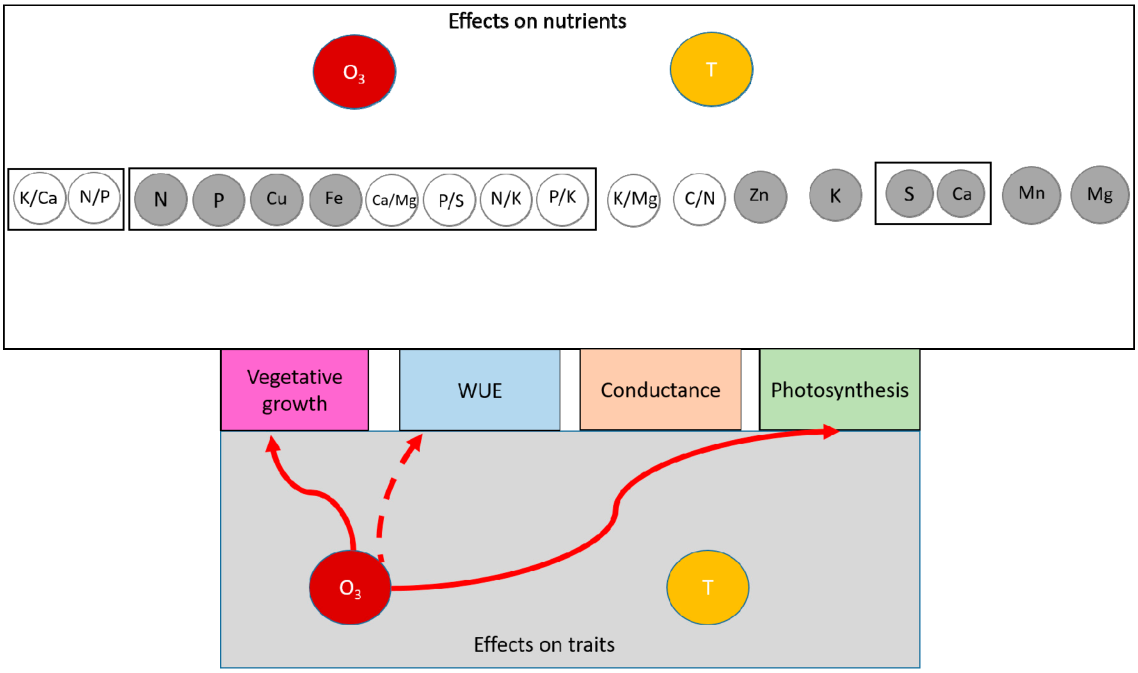

Figure S5. Ozone and temperature observed effects on growth and gas exchange measured traits and nutrients in *F. iberica*. Upper box, effects on nutrients and nutrients rates leaf amounts: red and yellow arrows denote the effects on nutrients of ozone (O_3) and temperature (T), respectively; pink, blue and green denote the correlation of nutrients (grey circles) and nutrient rates (white circles) with the traits; solid arrows indicate positive relationships and dashed arrows indicate negative relationships. Grey box, observed effect on traits: red and yellow arrows denote the effects on nutrients of O_3 and T; straight and solid arrows indicate positive relationships and straight dashed arrows indicate negative relationships; curve solid and dashed arrows mark concave and convex trend, respectively.

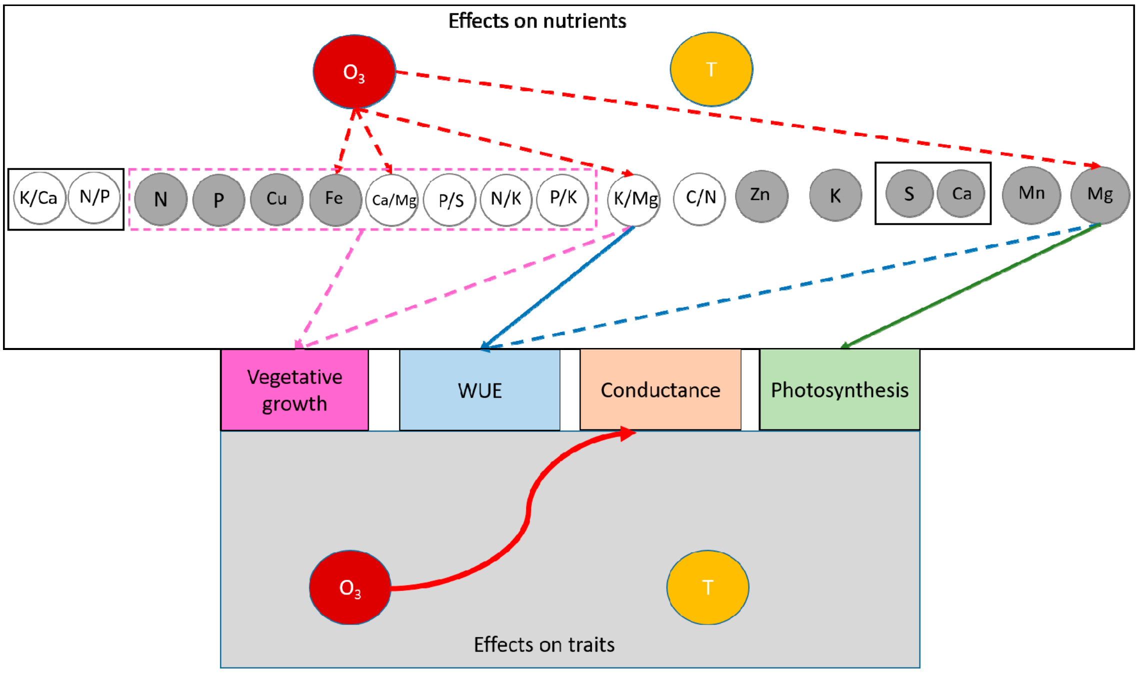

Supplement: Supplementary file 1 [file plants-12-00664-s001.zip › plants-2097782-supplementary.pdf]
